# How Can Cardiac Rehabilitation Promote Health Literacy? Results from a Qualitative Study in Cardiac Inpatients

**DOI:** 10.3390/ijerph19031300

**Published:** 2022-01-24

**Authors:** Anna Isselhard, Laura Lorenz, Wolfgang Mayer-Berger, Marcus Redaélli, Stephanie Stock

**Affiliations:** 1 Institute of Health Economics and Clinical Epidemiology, University Hospital of Cologne, 50935 Cologne, Germany; laura.lorenz@uk-koeln.de (L.L.); marcus.redaelli@uk-koeln.de (M.R.); stephanie.stock@uk-koeln.de (S.S.); 2 Centre for Cardiovascular Rehabilitation, 42799 Leichlingen, Germany; wolfgang.mayer-berger@klinik-roderbirken.de

**Keywords:** health literacy, cardiac rehabilitation, heart attack, empowerment

## Abstract

After acute care of a cardiac event, cardiac rehabilitation helps future disease management. Patients with low health literacy have been shown to have fewer knowledge gains from rehabilitation and higher all-cause mortality after acute cardiac events. Cardiac rehabilitation may be the best channel to target population with low health literacy, yet research on this topic is limited. Consequently, the main aim of the current study was to identify patient perceptions about the health literacy domains that are needed for successful rehabilitation of patients attending German cardiac rehabilitation clinics after an acute cardiac event. Five focus group interviews with 25 inpatients (80% male, 20% female) were conducted at a cardiac rehabilitation clinic in Germany. Patients were eligible to participate if they had sufficient understanding of the German language and had no other debilitating diseases. Patients identified five domains of health literacy for rehabilitation success: knowledge about their health condition; being able to find and evaluate health-related information, being able to make plans and sticking to them, assumption of responsibility over one’s health and the ability to ask for and receive support. The results give an important insight into what patients perceive as important components of their cardiac rehabilitation, which can provide the basis for developing the health literacy of patients and how cardiac rehabilitation clinics respond to the recovery needs of their patients.

## 1. Introduction

Cardiovascular diseases such as coronary heart disease remain the number one cause of death globally, with close to 18 million deaths every year [1] and health-care costs of roughly 111€ billion just within the European Union [2]. The development of cardiovascular diseases can be linked to non-modifiable risk factors, such as family history, age, gender, or ethnicity [3]. Modifiable risk factors, such as unhealthy behavioral habits like cigarette smoking, a diet high in fat and sugar, and a lack of physical exercise have also been found to be strongly linked to its genesis [3]. There are also a variety of psychosocial risk factors that have been linked to cardiovascular disease, such as chronic stress, low levels of education and low income, or health literacy challenges [4,5,6].

In its infancy, health literacy has been defined as the skill to read and comprehend written medical information, a concept now known as functional health literacy [7]. Research nowadays has adopted a broader scope of the term: it is generally agreed upon that health literacy encompasses all abilities to understand, evaluate and apply health information in order to navigate the healthcare system and to make conscious decisions and subsequently to stay both physically and mentally healthy and subsequently maintain or increase quality of life [7,8]. This includes not only being able to locate health information, reading comprehension and numeracy, but also being able to communicate and understanding physicians’ instructions and applying them. Accordingly, a person with health literacy challenges is often not able to access healthcare, read or understand basic information about health and illness, to communicate symptoms to their physician, to comprehend what they are being told to do, and to adhere to those instructions.

It has been shown that low levels of health literacy reliably predict detrimental health outcomes, such as lower uptake of preventive care [9], lower treatment adherence when in care [10], higher emergency room costs [11], and higher frequency of hospitalization [12,13]. In patients with cardiovascular diseases specifically, it has been shown that low health literacy is associated with lower disease-related knowledge [14,15], less well controlled blood pressure, less self-management behaviors, such as weight-monitoring, exercise behaviors, and salt consumption, as well as lower quality of life [15]. Two studies have shown that among patients with heart failure, those with low health literacy had a significantly higher all-cause mortality rates over the course of one year after hospitalization compared to those with adequate health literacy [16,17].

It is apparent that cardiovascular patients with low health literacy seem to have specific needs that are often not addressed in usual care. One channel to target this population is through cardiac rehabilitation. While originally created to recover patients from an acute cardiac event through exercise in the early 20th century, cardiac rehabilitation nowadays can be defined as a combination of medical and psychosocial interventions to assist patients with chronic or post-acute heart diseases [18,19].

In Germany, rehabilitation is usually initiated directly or shortly after acute care of a cardiac event, such as a heart attack or bypass surgery. It may be initiated by the hospital that provides acute care to the patient or through the patients’ primary care provider or cardiologist. Since 1974, rehabilitation is guaranteed to patients by German law [18].

The program during rehabilitation typically consists of a three-week exercise and diet regimen, combined with psychological services and education under the supervision of a professional team of physicians, nurses, psychologists, physiotherapists, and dieticians. Special attention is paid to the imparting of complex medical content about the genesis of cardiovascular issues and prevention of further cardiac events in order to trigger behavioral changes. The majority of rehabilitation services take place in an inpatient form; however, outpatient services have recently been added to complement the existing structures [19]. The distribution among genders in German cardiac rehabilitation is heavily skewed, with men constituting approximately 75–80% of all patients consistently from 1990 to 2016 [18,19,20]. To support patients well, cardiac rehabilitation services need to be able to respond to the health literacy needs of their patients, which means understanding those needs from the patient perspective.

In Germany, the “National Action Plan Health Literacy” (NAP for HL) was initiated as a scientific guideline to strengthen health literacy on an individual and systemic level [21]. Given the weighty impact of health literacy on the genesis of chronic disease and the management thereof, it is advisable to consider tailoring health care services, such as cardiac rehabilitation, to the health literacy level of the patient. There is evidence that patients with low health literacy levels have lower disease related knowledge, but also fewer knowledge gains in rehabilitation when compared to patients with adequate or high health literacy levels [22]. In other words, patients who would need to benefit from rehabilitation the most, take away far less than patients who are already able to locate, understand and apply health information. This in turn may lead to higher readmissions, higher health care costs and ultimately higher mortality for those patients with lower health literacy.

There are multiple solutions to this problem. Firstly, education in rehabilitation clinics could just assume that all patients have health literacy challenges. However, this could result in patients with adequate health literacy levels being uninterested and not taking away the maximum of knowledge they could have. Additionally, this form of rehabilitation care is not the most cost-efficient. A second approach may be to identify in which areas of health literacy patients face challenges and coach patients on an individual or group level with education more suited to their understanding. This has previously been shown to improve health outcomes in patients with cardiovascular disease with low health literacy [23]. Patient involvement in these studies appears to be limited.

While studies on how patients with various diseases conceptualize health literacy are available [24,25], studies on how cardiac rehabilitation inpatients identify the domains of health literacy they deem important for the purpose of cardiac rehabilitation are currently lacking from the literature. This has also been pointed out by the American Heart Association (AHA), that recommended high levels of patient involvement in care as well as scientific research [26]. In fact, many recommendations are congruent between the AHA and the NAP for HL in Germany. Both recommend a high level of patient activation and participation in all areas of the health care system.

Therefore, the aim of this study is to identify patient perceptions about the health literacy domains that are needed for successful rehabilitation of patients attending German cardiac rehabilitation clinics after an acute cardiac event. This has the potential to advance health literacy development in chronically ill populations by improving the understanding of the patient perspective. Examining this perspective has crucial implications as to how best design and deliver interventions in cardiac rehabilitation inpatients to address health literacy challenges.

## 2. Materials and Methods

### 2.1. Study Design

A non-experimental, qualitative focus group study was conducted in cardiac rehabilitation inpatients to elicit their views on which domains of health literacy were most important for a successful rehabilitation.

### 2.2. Interview Guide

The semi-structured interview guide was informed by the health literacy pathway model by Edwards and colleagues [25]. This model was adopted as a theoretical framework for the construct of health literacy to this study because it was specifically developed to explain health literacy in long-term health conditions. The model elaborates health literacy across five distinct stages: health knowledge, health literacy skills and practices, health literacy actions, production of informed options and informed decision-making. The interview guide was developed to cover all five stages of the model with 11 questions overall (see Supplementary Materials). Two stages (health literacy actions and informed-decision making) were discussed with the help of one scenario. Each scenario described a dilemma that patients could be confronted with after rehabilitation. The scenarios were designed to elicit required skills and domains of health literacy that were necessary to solve the dilemma, as perceived by the patients.

### 2.3. Setting

All interviews were held in the same rehabilitation clinic in Leichlingen, North Rhine-Westphalia, Germany. While Leichlingen is a more rural town, the catchment area of the clinic is the largest metropolitan area of Germany with a population of over 8 million people within a 50 km radius, encompassing not only big cities like Cologne (population: 1 million) or Duesseldorf (population: 600.000) but also very rural areas.

### 2.4. Recruitment

Since regular cardiac rehabilitation in Germany takes three weeks, patients were recruited in week two and the interviews were held in week three. The head physician at the clinic selected patients who matched the inclusion and exclusion criteria. A purposive sampling approach was adopted to ensure a diverse sample in terms of age, education, and migration background. Both male and female patients whose rehabilitation is paid for by the pension insurance fund “Rentenversicherung Rheinland” were eligible to be included. Because of the nature of this insurance fund, all patients were below the age of 65. Patients were not eligible to participate if they had no sufficient understanding of the German language and had other debilitating diseases, such as life-threatening cancer, dementia, or severe mental illnesses. Patients who were eligible were invited to a brief recruiting appointment, where they were told what to expect from the focus group interviews. After that, they had the opportunity to ask questions about the nature of the study. If they agreed to participate, they signed consent forms and were invited to a focus group interview in the following week. The patients were not incentivized for participating. Recruitment was arranged weekly on a rolling basis until theoretical saturation was reached, meaning that no new domains of health literacy could be identified in a preliminary analysis immediately after the interviews.

### 2.5. Procedure

The same two researchers (A.I. and L.L.) conducted all focus group interviews. The researchers are both experienced in conducting and analyzing qualitative research. The researchers were unknown to the patients prior to the interviews and were not involved in the care of patients thereafter. The main goal of the researchers was to allow every participant to speak their mind and to facilitate communication. To begin the conversation, patients were asked about their experiences in rehabilitation and the skills they feel they needed in rehabilitation and for navigating a healthy life after rehabilitation. To facilitate the conversation, the researchers asked the participants to identify skills that were necessary to handle the situations described in the scenarios.

All focus group discussions were audiotaped with the same voice recorder and with the permission of the participants. The audiotapes were turned on after the introduction round to ensure that no statements given could be traced back to any identifying information. After every focus group interview, the audiotape was transferred to a password protected computer and subsequently deleted from the voice recorder. A third-party transcription bureau performed the transcription of the audiotapes.

### 2.6. Analysis

The transcriptions were coded by two independent researchers (A.I. and L.L.) along an open coding scheme using qualitative content analysis by Mayring [27] via the software MAXQDA (VERBI GmbH, Berlin, Germany). Qualitative content analysis aims at classifying qualitative data into categories of similar connotation. Conflicts in coding were resolved by discussion among A.I., L.L., S.S.T., and M.R.

### 2.7. Ethical Considerations

The focus group interviews were carried out in accordance with the Declaration of Helsinki. The potential risk to focus group participants was reduced to a minimum by obtaining informed consent prior to the interview and by not discussing identifying information on the audio recordings. The University of Cologne ethics committee reviewed and approved the focus group protocols.

## 3. Results

Overall, 25 cardiac rehabilitation inpatients (20 male, five female) participated in the focus group interviews. Five patient focus group interviews were conducted. The patients ranged in age from 32 to 64 years with a mean of 55 years in female patients (age range: 46–63 years) and a mean of 52.4 years in male patients (age range: 32–64 years). The focus groups ranged in length from 45 to 100 min with a mean of 76 min. One hundred and ninety-seven single-spaced pages were included in the analysis.

The analysis of the focus group interviews resulted in the identification of five skills, that patients saw as the main domains of health literacy for cardiac rehabilitation: health-related knowledge, information-seeking, self-regulation, assumption of responsibility and communication/interactive skills. The results for each component of health literacy from the patients’ perspective are presented with demonstrative quotes from the patients.

### 3.1. Identified Domains of Health Literacy

#### 3.1.1. Knowledge

Our analysis showed that patients from all focus groups identified knowledge about cardiac events, illness, medication as well as healthy behaviors that will prevent future cardiac events as the most significant domain of health literacy for rehabilitation success. Knowledge was mentioned first in every single focus group interview, stressing the importance of having a solid understanding of disease and healthy living. Patients especially emphasized the need to get information suited to their needs, their own medical history and specifically, tailored to their level of understanding. Two patients noted:

“*Knowledge about your condition and understanding is the utmost basis to take anything away from rehabilitation.*”

(FG01-03, male patient, focus group 1)

“*I should not have to go to medical school to understand my condition, but I should be able to understand, just for myself, what happened to me and where.*”

(FG03-02, male patient, focus group 3)

#### 3.1.2. Information-Seeking

While patients agreed that gathering and understanding health-related information was a crucial aspect of health literacy, they specified that identifying which sources for health information were reliable would be vital in this process. Most patients specifically experienced difficulties judging the dependability with information found on the internet and pointed out that learning about reliable sources would be an important step for rehabilitation and healthy living. Patients would often consult their primary care physician after finding information online.

“*There are renowned websites, where you can certainly find information. […] I think you need to be very careful, where you get your information.*”

(FG01-05, male patient, focus group 1)

“[After searching online] *I would ask my doctor, if the information are actually true. My doctor also said to me “Don’t ask Dr. Google, ask me*”

(FG05-01, female patient, focus group 5)

“*You probably can’t trust everything you read online about your condition. But it can be difficult to judge which information are reliable*”

(FG04-02, male patient, focus group 4)

#### 3.1.3. Self-Regulation

Patients agreed that forming intentions and upholding motivation after rehabilitation would be crucial to not be readmitted and therefore identified self-regulation an important aspect of health literacy. The term self-regulation encompasses the ability to form intentions, stay motivated and exert self-control. Despite identifying this component, some patients expressed doubts about their self-regulation skills in order to navigate a healthy lifestyle.

“*I know myself–I always have good and strong intentions. I hope I can stick to them this time.*”

(FG05-05, female patient, focus group 5)

“*It is the inner couch potato that needs to be fought.*”

(FG03-04, male patient, focus group 3)

“*I think setting goals, devising plans and so forth are very important in the next few months. In the past, I would always say “I’ll do it tomorrow”, then “next week” and then you are lost. Hopefully, this *[healthy living]* will become a habit.*”

(FG02-02, male patient, focus group 2)

#### 3.1.4. Assumption of Responsibility

Most patients agreed that in order to care for one’s health, one would first have to assume responsibility over one’s health. Some patients admitted that before experiencing an acute cardiac event, they believed that health was likely the result of genetic advantages of some people or luck of the draw. Several patients reported that only after experiencing a “wake-up call”, they now think that assuming responsibility over one’s health is beneficial in making health-related decisions and for continued rehabilitation success.

“*All these risk factors, that I knew of deep down, I just ignored them.* […] *Now I am extremely aware of my responsibility.*”

(FG02-05, male patient, focus group 2)

“[This is] *what I meant–gaining consciousness. I have never even thought about my health before*.”

(FG04-01, male patient, focus group 4)

“*Diet, exercise and smoking–these things are really up to myself. No one else. And now the doctor in the hospital said: “Don’t look back”, because it is up to me to move on and take control.*”

(FG03-03, male patient, focus group 3)

#### 3.1.5. Communication/Interactive Skills

Patients report that interactive skills with their health care providers and their social environment are crucial parts of health literacy. More specifically, patients agreed that communication with their health care providers and asking their social environment for support are an important aspect of health literacy and tremendously help rehabilitation success. While this skill overall was described by both male and female patients, the need for social support was more frequently mentioned by male patients.

“*I always think having my wife sit next to me* [in rehabilitation], *it would have double the effect.*”

(FG01-03, male patient, focus group 1)

“*I never asked for help and always said “yes” to everything*. […] *but sometimes I need help, too*”

(FG05-04, female patient, focus group 5)

“*I believe that the communication with your physician plays an important role and that you trust them with everything.*”

(FG01-06, male patient, focus group 1)

## 4. Discussion

This qualitative study aimed at identifying and presenting components of health literacy for German cardiac rehabilitation inpatients from the patients’ perspective. For this purpose, focus group interviews were carried out with patients from a German cardiac rehabilitation clinic. A thorough analysis of the collected data based on qualitative content analysis revealed that patients identified five domains of health literacy for rehabilitation success: knowledge about their health condition and healthy living (knowledge); being able to find and evaluate health-related information (information-seeking); being able to make plans and sticking to them (self-regulation); taking on responsibility over one’s health (assumption of responsibility); and the ability to ask for and receive support (communication/interactive skills). Thus, patients agreed that in order to have rehabilitation success one would need to: (1) know about their condition and health in general, (2) to know where to look for and how to evaluate health information, (3) be able to make behavioral plans and uphold motivation to follow them, (4) take on the responsibility for one’s health, and (5) have appropriate interactive skills to express needs for social support to their social environment as well as to express symptoms to their primary care physicians.

Many of those patient-identified domains have previously been included in various health literacy models. Knowledge has been included in virtually all health literacy definitions, including the comprehensive definition by Sørenson et al. [28] which comprises several different health literacy definitions in the literature.

Locating health-related information is also a very prominent component of health literacy models. In fact, the most used definition of health literacy states includes accessing and understanding health information [8]. The model by Nutbeam proposed that obtaining health information represents the base of functional health literacy, the lowest level of the stage model of health literacy [6].

Interactive skills, as defined by the patients in this study, can be compared to interactive health literacy as defined by Nutbeams stage model of health literacy [29]. According to this model, interactive health literacy refers to skills that can be used to stimulate self-help by communication, for example by more fruitful interactions with health care providers or the social environment.

Assumption of responsibility is not as prominent in health literacy models as knowledge or interactive skills, but a few definitions and models include the importance of people accepting responsibility over their own health, such as the health literacy definition by Kickbusch et al. [28] or the health literacy model by Rudinger [29]. In the latter, assumption of responsibility counts as an advanced psychosocial skill that in turn promotes better self-regulatory skills.

Finally, self-regulation has a long-standing history in research of health behavior on its own [30]. Previous research has found that while most people want to live a healthy lifestyle, many fail to form clear goals and strive for goal achievement. In the health literacy field, sufficient self-regulation has at times been listed as a component of health literacy [29], while other models propose that good self-regulation would be an outcome from sufficient health literacy [6,8]. To date, it is not generally agreed upon whether self-regulation is a part of health literacy or a consequence thereof.

Our results compare with longitudinal qualitative studies with patients with cardiovascular disease in Ireland that investigated improvements in health literacy after an outpatient cardiovascular risk reduction program that has similar content to German cardiac rehabilitation [31,32]. Patients in this study identified most improvements in control over their health and self-management, which are similar in meaning to assumption of responsibility and self-regulation identified in our study [32]. Furthermore, patients reported improved knowledge on their health condition as well as improvement with information-seeking.

Our study has several limitations that need to be addressed. First of all, even though it was intended to include patients from diverse backgrounds, self-selection bias cannot entirely be avoided. This means that patients who volunteered to participate in the focus group interviews were perhaps the patients who possess a certain degree of health literacy and had a good idea about what skills were necessary. Secondly, the researchers decided to conduct male and female interviews separately from each other after observing a high rate of refusal from female patients to participate in mixed focus groups. This could have multiple reasons, but the most likely reason seems to be that the portion of female patients is much lower than the portion of male patients, and that female patients did not want to disclose information in front of male patients. The researchers decided to add a female-only focus group interview, in which there were no refusals to participate. This further supports the notion of female patient refusal due to privacy reasons in an otherwise male dominated group. However, we cannot exclude that our results could have been slightly different in mixed groups due to greater heterogeneity.

Another limitation that needs to be addressed is the timing of the focus group interview. As described above, patients were recruited in week two of three and interviews were conducted in the final week of cardiac rehabilitation. Therefore, patients arguably were more knowledgeable as they would have been had interviews been conducted at the beginning of the rehabilitation. For future research, it would be worth-while to compare results from a more naïve sample to our results.

## 5. Conclusions

The integration of the qualitative results of this study into previous research demonstrates that the majority of domains identified by patients in cardiac rehabilitation are already part of health literacy definitions. It is interesting to observe that while many definitions of health literacy in the literature did not specifically include the patient perspective, the majority of domains are congruent with patient-identified domains. A key finding from this study is that patients included domains such as assumption of responsibility and self-regulation, which are not consistently included in health literacy models. These specific domains, in turn, have been included in other studies that have specifically asked patients for their insights. The identified domains of health literacy are currently not all sufficiently addressed in cardiac rehabilitation. An integration of these domains into cardiac rehabilitation should be considered as a focus on the integration of learned contents into daily life may be beneficial for patient outcomes.

On a broader level, the results of our study further the development of health literacy in chronic disease in general, as understanding health literacy from the patient perspective in chronically ill populations should be the first step in developing interventions and improving clinical care.

## Data Availability

The dataset used and/or analyzed during this study are available from the corresponding author upon reasonable request.

## Supplementary Materials

The following are available online at https://www.mdpi.com/article/10.3390/ijerph19031300/s1, Semi-structured interview guide.

## References

[1] World Health Organization Fact Sheet: Cardiovascular Diseases (CVDs). https://www.who.int/en/news-room/fact-sheets/detail/cardiovascular-diseases-(cvds).

[2] Wilkins E., Wilson L., Wickramasinghe K., Bhatnagar P., Leal J., Luengo-Fernandez R., Burns R., Rayner M., Townsend N. (2017). European Cardiovascular Disease Statistics 2017.

[3] Virani S.S., Alonso A., Benjamin E.J., Bittencourt M.S., Callaway C.W., Carson A.P., Chamberlain A.M., Chang A.R., Cheng S., Delling F.N. (2020). Heart Disease and Stroke Statistics—2020 Update: A Report from the American Heart Association. Circulation.

[4] Winkleby M.A., Jatulis D.E., Frank E., Fortmann S.P. (1992). Socioeconomic status and health: How education, income, and occupation contribute to risk factors for cardiovascular disease. Am. J. Public Health.

[5] Safeer R.S., Cooke C.E., Keenan J. (2006). The impact of health literacy on cardiovascular disease. Vasc. Health Risk Manag..

[6] Nutbeam D. (2000). Health literacy as a public health goal: A challenge for contemporary health education and communication strategies into the 21st century. Health Promot. Int..

[7] Nutbeam D. (2008). The evolving concept of health literacy. Soc. Sci. Med..

[8] Sørensen K., Van den Broucke S., Fullam J., Doyle G., Pelikan J., Slonska Z., Brand H., (HLS-EU) Consortium Health Literacy Project European (2012). Health literacy and public health: A systematic review and integration of definitions and models. BMC Public Health.

[9] Scott T.L., Gazmararian J.A., Williams M.V., Baker D.W. (2002). Health Literacy and Preventive Health Care Use Among Medicare Enrollees in a Managed Care Organization. Med. Care.

[10] Kalichman S.C., Ramachandran B., Catz S. (1999). Adherence to combination antiretroviral therapies in HIV patients of low health literacy. J. Gen. Intern. Med..

[11] Howard D.H., Gazmararian J., Parker R.M. (2005). The impact of low health literacy on the medical costs of Medicare managed care enrollees. Am. J. Med..

[12] Berkman N.D., Sheridan S.L., Donahue K.E., Halpern D.J., Crotty K. (2011). Low Health Literacy and Health Outcomes: An Updated Systematic Review. Ann. Intern. Med..

[13] Baker D.W., Parker R., Williams M.V., Clark W.S. (1998). Health literacy and the risk of hospital admission. J. Gen. Intern. Med..

[14] Williams M.V., Baker D.W., Parker R.M., Nurss J.R. (1998). Relationship of Functional Health Literacy to Patients’ Knowledge of Their Chronic Disease. Arch. Intern. Med..

[15] Macabasco-O’Connell A., DeWalt D., Broucksou K.A., Hawk V., Baker D.W., Schillinger D., Ruo B., Bibbins-Domingo K., Holmes G.M., Erman B. (2011). Relationship Between Literacy, Knowledge, Self-Care Behaviors, and Heart Failure-Related Quality of Life Among Patients with Heart Failure. J. Gen. Intern. Med..

[16] McNaughton C.D., Cawthon C., Kripalani S., Liu D., Storrow A.B., Roumie C.L. (2015). Health Literacy and Mortality: A Cohort Study of Patients Hospitalized for Acute Heart Failure. J. Am. Heart Assoc..

[17] Peterson P.N. (2011). Health Literacy and Outcomes Among Patients with Heart Failure. JAMA J. Am. Med. Assoc..

[18] Karoff M., Held K., Bjarnason-Wehrens B. (2007). Cardiac rehabilitation in Germany. Eur. J. Cardiovasc. Prev. Rehabil..

[19] Mittag O., Schramm S., Böhmen S., Hüppe A., Meyer T., Raspe H. (2011). Medium-term effects of cardiac rehabilitation in Germany: Systematic review and meta-analysis of results from national and international trials. Eur. J. Cardiovasc. Prev. Rehabil..

[20] Bellmann B., Lin T., Greissinger K., Rottner L., Rillig A., Zimmerling S. (2020). The Beneficial Effects of Cardiac Rehabilitation. Cardiol. Ther..

[21] Schaeffer D., Hurrelmann K., Bauer U., Kolpatzik K. (2018). Nationaler Aktionsplan Gesundheitskompetenz. Die Gesundheitskompetenz in Deutschland Starken.

[22] Mattson C.C., Rawson K., Hughes J.W., Waechter N., Rosneck J. (2014). Health literacy predicts cardiac knowledge gains in cardiac rehabilitation participants. Heal. Educ. J..

[23] Lee T.W., Lee S.H., Kim H.H., Kang S.J. (2012). Effective Intervention Strategies to Improve Health Outcomes for Cardiovascular Disease Patients with Low Health Literacy Skills: A Systematic Review. Asian Nurs. Res..

[24] Jordan J.E., Buchbinder R., Osborne R. (2010). Conceptualising health literacy from the patient perspective. Patient Educ. Couns..

[25] Edwards M., Wood F., Davies M., Edwards A. (2012). The development of health literacy in patients with a long-term health condition: The health literacy pathway model. BMC Public Health.

[26] Magnani J.W., Mujahid M.S., Aronow H.D., Cené C.W., Dickson V.V., Havranek E., Morgenstern L.B., Paasche-Orlow M.K., Pollak A., Willey J.Z. (2018). Health Literacy and Cardiovascular Disease: Fundamental Relevance to Primary and Secondary Prevention: A Scientific Statement from the American Heart Association. Circulation.

[27] Mayring P. (2003). Qualitative Inhaltsanalyse—Grundlagen und Techniken [Qualitative Content Analysis—Basics and Techniques].

[28] Kickbusch I., Wait S., Maag D. (2006). Navigating health: The role of health literacy. London: Alliance for Health and the Future.

[29] Lenartz N., Soellner R., Rudinger G.G. (2014). Modellbildung und empirische Modellprüfung einer Schlüsselqualifikation für gesundes Leben. DIE Z. für Erwachs..

[30] De Ridder D.T.D., De Wit J.B.F. (2006). Self-regulation in health behaviour: Concepts, theories, and central issues. Self-Regulation in Health Behaviour.

[31] McKenna V.B., Sixsmith J., Barry M.M. (2018). A Qualitative Study of the Development of Health Literacy Capacities of Participants Attending a Community-Based Cardiovascular Health Programme. Int. J. Environ. Res. Public Health.

[32] McKenna V.B., Sixsmith J., Barry M. (2020). Facilitators and Barriers to the Development of Health Literacy Capacities Over Time for Self-Management. HLRP Health Lit. Res. Pract..

