# Supplementary material for: How Can Cardiac Rehabilitation Promote Health Literacy? Results from a Qualitative Study in Cardiac Inpatients
Source: Int J Environ Res Public Health. 2022 Jan 24;19(3):1300. doi: 10.3390/ijerph19031300 (PMC8835102; doi:10.3390/ijerph19031300)
Supplement: Supplementary file 1 [file ijerph-19-01300-s001.zip › ijerph-1464381-supplementary.pdf]

Appendix – Focus group guideline

Phase	Description	Main question and scenario	Follow-up questions and probes
Introduction	• Introduction of interviewers<br>• Answer any open questions<br>• Introduction round of participants	-	-
Beginning of audio recording			
Warm-up	A warm-up phase was included to make patients more comfortable to share their thoughts in front the group	• How was your experience in cardiac rehabilitation so far? In your mind, what is important for a successful rehabilitation?<br>• What did you learn in rehabilitation so far that you think will be helpful in the future?	• What did you like about it?<br>• What could be improved?
Main Interview (domains based on Edwards [24].)	Health knowledge	• What did you learn about cardiovascular disease/healthy living?<br>• How important is being knowledge about your disease to you?	• What about this was new to you?<br>• Why is this important/not important to you?
	Health literacy skills and practices	• How do you go about getting information about your disease/healthy living?	• Where do you look?<br>• How do you judge validity of information?
	Health literacy actions	Scenario: Imagine coming home from a busy day at work. You know that you are supposed to work out for 30 minutes. But you feel tired, stressed, and hungry. Maybe your partner wants to order food and have a relaxed night, your children need help with their homework, or your favorite soccer team is playing.<br>• How do you imagine behaving in this situation?<br>• What could help you get your work out done?	• Which skills are needed?<br>• Social support?<br>• Digital support (apps, reminder calls)?
	Production of Informed Options	• Are you currently using medication for disease management? If so, do you know what they are supposed to do?<br>• Do you know if there are alternative medication or treatments?	• How important is it to you to know if there are alternative medications or treatments?
	Making a Shared Decision	Scenario: Imagine speaking with your pharmacist about an unpleasant side effect. They recommend an alternative.<br>• Do you bring this up to your doctor? If so, how confident are you in this situation?<br>• How important is it to you to be involved in treatment decisions?	• Which skills can you identify for a scenario like this?
